# Therapeutic Delivery of rAAV *sox9* via Polymeric Micelles Counteracts the Effects of Osteoarthritis-Associated Inflammatory Cytokines in Human Articular Chondrocytes

**DOI:** 10.3390/nano10061238

**Published:** 2020-06-25

**Authors:** Jonas Urich, Magali Cucchiarini, Ana Rey-Rico

**Affiliations:** 1 Center of Experimental Orthopaedics, Saarland University Medical Center, D-66421 Homburg, Germany; jonas.urich@gmx.de (J.U.); mmcucchiarini@hotmail.com (M.C.); 2 Cell Therapy and Regenerative Medicine Unit, Centro de Investigacións Científicas Avanzadas (CICA), Universidade da Coruña, ES-15071 A Coruña, Spain

**Keywords:** osteoarthritis, human articular cartilage, rAAV vectors, SOX9, polymeric micelles, pro-inflammatory cytokines, IL-1β, TNF-α

## Abstract

Osteoarthritis (OA) is a prevalent joint disease linked to the irreversible degradation of key extracellular cartilage matrix (ECM) components (proteoglycans, type-II collagen) by proteolytic enzymes due to an impaired tissue homeostasis, with the critical involvement of OA-associated pro-inflammatory cytokines (interleukin 1 beta, i.e., IL-1β, and tumor necrosis factor alpha, i.e., TNF-α). Gene therapy provides effective means to re-establish such degraded ECM compounds by rejuvenating the altered OA phenotype of the articular chondrocytes, the unique cell population ubiquitous in the articular cartilage. In particular, overexpression of the highly specialized SOX9 transcription factor via recombinant adeno-associated viral (rAAV) vectors has been reported for its ability to readjust the metabolic balance in OA, in particular via controlled rAAV delivery using polymeric micelles as carriers to prevent a possible vector neutralization by antibodies present in the joints of patients. As little is known on the challenging effects of such naturally occurring OA-associated pro-inflammatory cytokines on such rAAV/polymeric gene transfer, we explored the capacity of polyethylene oxide (PEO) and polypropylene oxide (PPO)-based polymeric micelles to deliver a candidate rAAV-FLAG-h*sox9* construct in human OA chondrocytes in the presence of IL-1β and TNF-α. We report that effective, micelle-guided rAAV *sox9* overexpression enhanced the deposition of ECM components and the levels of cell survival, while advantageously reversing the deleterious effects afforded by the OA cytokines on these processes. These findings highlight the potentiality of polymeric micelles as effective rAAV controlled delivery systems to counterbalance the specific contribution of major OA-associated inflammatory cytokines, supporting the concept of using such systems for the treatment for chronic inflammatory diseases like OA.

## 1. Introduction

Osteoarthritis (OA) represents a prevalent, chronic, and deteriorating joint affliction that is the leading cause of impaired function and disability [1]. OA is characterized by multiple functional and structural cartilage tissue and cell shifts, such as the progressive and permanent degradation of the articular cartilage matrix (loss of type-II collagen and of proteoglycans), the restructuration of the subchondral bone, and the formation of osteophytes [2,3] due to defective homeostasis [4,5]. Of note, none of the current pharmacological options and surgical alternatives [1] for treating OA can reestablish the native cartilage quality in patients.

Current research associates the changes observed in OA disease with a complex cascade of biochemical factors, including proteolytic enzymes that promote the disruption of the cartilage macromolecules [6]. Pro-inflammatory cytokines such as interleukin 1 beta (IL-1β) and tumor necrosis factor alpha (TNF-α), produced by mononuclear cells, activated synoviocytes or by the cartilage itself, upregulate metalloproteinases gene expression, impairing chondrocyte counteracting synthetic pathways necessary to reinstate the integrity of the degenerated extracellular matrix (ECM) [6].

In this context, previous studies have shown an abolishment of type-II collagen expression from primary human articular chondrocytes via suppression of the expression of the cartilage-associated sex-determining region Y-type high mobility box 9 (SOX9) transcription factor upon treatment with IL-1β [7]. Of note, overexpression of *sox9* via lentiviral vector has already been shown to preserve chondrocytes from IL-1β-induced apoptosis and degeneration [8]. However, while efficient, lentiviral vectors are not well adapted for translational approaches, as they involve a risk of insertional mutagenesis upon integration into the genome of host cells [9]. In contrast, recombinant adeno-associated viral (rAAV) vectors mainly remain episomal in the nucleus of their targets, showing potential integration events at very low frequency (0.1–1% vide infra) [10], while also allowing for highly effective gene transfer efficiencies even in nondividing cells like articular chondrocytes (more than 70%) [11]. rAAV vectors have thus emerged as the preferred gene carriers in several regenerative medicine applications including for cartilage repair [12,13,14,15,16].

A high and prolonged gene transmission efficiency in articular chondrocytes both in vitro and through their compact ECM in situ has been reported via rAAV vectors (up to 80% for at least 150 days) has been reported [11]. Furthermore, gene transfer of an rAAV TGF-β vector has been shown to promote the biological activities both in human articular chondrocytes cultures in vitro and in articular cartilage explants in situ [17,18]. In addition, overexpression of *sox9* via rAAV led to increased levels of type-II collagen and proteoglycans in both normal and OA-affected articular chondrocytes in vitro [19].

Still, administration of rAAV vectors in patients may be hampered by the prevalence of anti-AAV antibodies directed against viral capsid proteins in individuals as those prevailing in synovial fluid from patients affected with joint disorders [20]. We previously described the suitability of rAAV vectors (*lacZ*) encapsulation in poly(ethylene oxide) (PEO) and poly(propylene oxide) (PPO)-based polymeric micelles from linear (poloxamers; PF68) or X-shaped copolymers (poloxamines; T908), as a way to overcome such obstacles while affording protection to the vectors in experimental settings of neutralization and increasing their gene transfer efficacy [21,22]. Interestingly, overexpression of *sox9* using such systems resulted in the effective remodeling of human OA cartilage, leading to increases in cell proliferation activities and in proteoglycan deposition relative to free vector administration [23]. Yet, it remains to be seen whether such micellar systems can also be efficient for delivering rAAV vectors and overexpressing their transgenes in an inflammatory, detrimental environment like in OA (IL-1β, TNF-α) [4,5,24].

The aim of the present study was therefore to test the ability of PF68- and T908-based polymeric micelles to deliver the therapeutic rAAV-FLAG-h*sox9* candidate vector in human OA chondrocytes, the sole cell population present in the articular cartilage, in the presence of OA-associated pro-inflammatory cytokines (IL-1β, TNF-α) in a 2D environment as a preliminary proof of concept, as a means to effectively restore the chondrocyte phenotype in such cells in vitro.

## 2. Materials and Methods

### 2.1. Materials

Pluronic^®^ F68 and Tetronic^®^ 908 were generously provided by BASF (Ludwigshafen, Germany). The pro-inflammatory cytokines (IL-1β, TNF-α) were obtained from Prepotech (Hamburg, Germany). The anti-SOX9 (C-20) antibody was purchased at Santa Cruz Biotechnology (Heidelberg, Germany) and the anti-type-II collagen (II-II6B3) antibody at DSHB (Iowa, IA, USA). Biotinylated secondary antibodies and the ABC reagent were obtained from Vector Laboratories (Alexis Deutschland GmbH, Grünberg, Germany). Alcian blue 8GX was from Sigma (Munich, Germany). The Cell Proliferation Reagent WST-1 was obtained from Roche Applied Science (Mannheim, Germany).

### 2.2. Cells

Human osteoarthritic (OA) cartilage (Mankin score 7–9) was obtained from total knee arthroplasty samples (n = 4) from patients, after informed consent signature [18] before inclusion in the study. The study was approved by the Ethics Committee of the Saarland Physicians Council (*Ärztekammer des Saarlandes*, reference number Ha06/08). All procedures were in conformity with the Helsinki Declaration. Human OA chondrocytes (passage 1–2) were isolated by collagenase digestion of cartilage slices as previously described [18,22] and cultured in DMEM, 10% FBS, 100 U/mL penicillin G, 100 µL/mL streptomycin (growth medium) prior to the studies, without cell dedifferentiation.

### 2.3. Plasmids and rAAV Vectors

rAAV-FLAG-h*sox9* is derived from pSSV9, an AAV-2 genomic clone [25,26], and carries a FLAG-tagged human *sox9* cDNA under the control of the cytomegalovirus immediate-early (CMV-IE) promoter [23,27,28,29]. The vectors were packaged using a helper-free, two-plasmid transfection system in 293 cells with the Adenovirus helper plasmid pXX6 and the packaging plasmid pXX2 [18]. The resulting vector preparations were extensively dialyzed and titrated by real-time PCR [18,30,31], averaging 10^10^ transgene copies/mL.

### 2.4. Preparation of Micellar Copolymer Solutions Containing rAAV-FLAG-hsox9 Vectors

Copolymer solutions (PF68 or T908) were prepared in 10% sucrose aqueous solution at 4 °C, mixed with rAAV-FLAG-h*sox9*, and maintained in ice-water bath for 30 min prior to their use as previously described [21,22,23]. The final micellar concentration into the culture medium was 2%. Effective interaction between the vectors and the polymeric micelles was confirmed by dynamic light scattering and electron microscopy [21,22,23].

### 2.5. Gene Transfer in Inflammatory Conditions via rAAV-FLAG-hsox9/Polymeric Micelles

Human OA chondrocytes (3000 cells/well or 40,000 cells/well for Alcian blue staining) were seeded in 96-well plates and maintained for 12 h at 37 °C under 5% CO_2_ as previously described [22,23]. Monolayer cultures of OA chondrocytes were directly transduced with the rAAV-FLAG-h*sox9*/polymeric micelles (2 × 10^8^ transgene copies, micellar concentration 2%) or after pre-incubation for 4 h with IL-1β (10 ng/mL) [32] or TNF-α (100 ng/mL) only [33], or concomitantly with IL-1β and TNF-α (10 and 100 ng/mL, respectively). Control conditions included cells cultured without vector treatment or copolymer solution (negative control) and cells transduced with free rAAV vector (positive control). Cultures were maintained for 10 days with 3 weekly medium changes.

Expression of SOX9 was monitored by immunocytochemistry using and anti-SOX9 specific primary antibody, a biotinylated secondary antibody, with the ABC method with diaminobenzidine (DAB) as previously described [23,29]. To control for secondary immunoglobulins, OA chondrocytes in monolayers cultures were assayed with exclusion of the primary antibody. All cultures were inspected under light microscopy (Olympus CKX41).

### 2.6. Histological and Immunocytochemical Analyses

Chondrocytes in monolayer cultures were harvested after 1 and 10 days and fixed in 4% formalin [21,22,23] prior to the immunocytochemical analyses. Expression of SOX9 and type-II collagen was detected using specific primary and biotinylated secondary antibodies, and the ABC method with DAB chromogen, with examination under light microscopy (Olympus CKX41) [21,22]. Alcian blue staining was involved to detect matrix proteoglycans [21,22,34,35]. Briefly, fixed monolayer cultures were stained with Alcian blue (1% in HCl 1 N) and excess stain was washed with double distilled water. The staining was solubilized by overnight incubation in 6 M guanidine hydrochloride and the absorbance at 595 nm was quantified with a GENios spectrophotometer (Tecan Crailsheim, Germany).

### 2.7. Histomorphometry

The mean intensities of SOX9 and type-II collagen immunostaining (ratio of positively stained surface to the total surface) were assessed at four randomized locations for each replicate condition as previously described [21,22,23]. Analyses were accomplished by using SIS AnalySIS (Olympus, Hamburg, Germany) and Adobe Photoshop (Adobe Systems Software CS2, Unterschleissheim, Germany) [21,23].

### 2.8. Evaluation of Cell Proliferation and Viability

Proliferation of chondrocytes in monolayer cultures was estimated using the Cell Proliferation Reagent WST-1, with optical density (OD) values proportional to the cell numbers [22,23,30]. Controls included the same conditions depicted in 2.5. ODs at 450 nm were registered using a GENios spectrophotometer (Tecan) and the percent’s of cell viability were calculated as follows:

Viability (%) = [(OD sample)/(OD negative control)] × 100(1)

### 2.9. Statistical Analysis

Each condition was tested in duplicate in four independent experiments using all patients. The values registered are depicted as mean ± standard deviation (SD). A *t*-test was employed, with *p* < 0.05 being considered statistically significant.

## 3. Results

### 3.1. Efficacy of rAAV-Mediated sox9 Overexpression in Conditions of Inflammation upon Vector Delivery via Polymeric Micelles

We first evaluated whether the presence of pro-inflammatory cytokines may alter the overexpression of SOX9 in human OA chondrocytes monolayer cultures.

**Figure 1:**
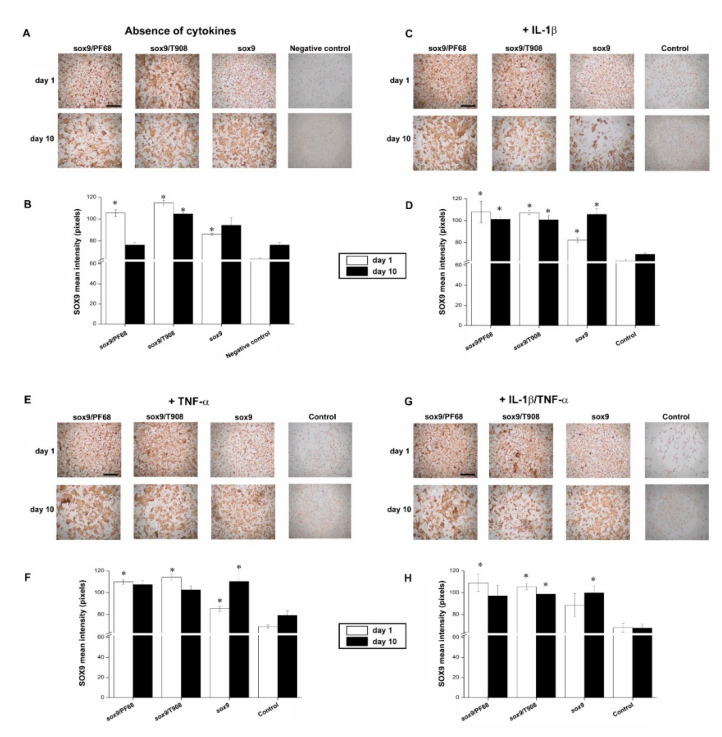
Transgene expression in rAAV-FLAG-h*sox9*-transduced human OA chondrocytes using polymeric micelles. Cells in mnolayer culture were directly transduced with the rAAV/polymeric micelles (**A**,**B**) or after pre-incubation for 4 h with IL-1β (10 ng/mL) (**C**,**D**), TNF-α (100 ng/mL) (**E**,**F**), or IL-1β/TNF-α (10/100 ng/mL) (**G**,**H**), as described in the Materials and Methods. The cultures were then processed after 1 and 10 days to detect SOX9 expression by immunocytochemistry (magnification x4, scale bar 500 µm; all representative data) (**A**,**C**,**E**,**G**) with corresponding histomorphometric analyses (**B**,**D**,**F**,**H**), as described in the Materials and Methods. Control conditions included the absence of copolymer or vector treatment (negative control) and the application of free rAAV vector (positive control). * Statistically significant compared with the negative control at similar time points.

In agreement with our previous observations [23], effective SOX9 overexpression was noted in the cells via rAAV-FLAG-hsox9 transduction (up to a 1.4-fold increase relative to the negative control in the absence of cytokines, *p* = 0.021) (Figure 1A,B). Similarly, supply of rAAV in polymeric micelles led to the most intense SOX9 immunoreactivity (up to a 1.8-fold increase when compared with the negative control in the absence of cytokines on day 1, *p* = 0.015), leading to more sustained levels of expression over time (up to a 1.4-fold difference with respect to the cell control in the absence of cytokines on day 10, *p* = 0.009) (Figure 1A,B).

Treatment with IL-1β did not alter the levels of SOX9 expression early on (*p* = 0.450 compared with the negative control in the absence of cytokines on day 1) (Figure 1A,B versus Figure 1C,D) while a reduction was noted after 10 days (up to a 1.1-fold difference with respect to the negative control in the absence of cytokines, *p* = 0.106) (Figure 1A,B versus Figure 1C,D). Interestingly, the treatment with rAAV-FLAG-hsox9 promoted a significant enhancement in SOX9 expression levels following IL-1β treatment (up to a 1.5-fold difference with respect to the cell control in the presence of IL-1β, *p* = 0.024), especially upon vector delivery via micellar systems (up to a 1.7-fold difference when compared with the cell control in the presence of IL-1β on day 1, *p* = 0.040) (Figure 1C,D). Such effects were also maintained over the time of evaluation (up to a 1.5-fold increase with respect to the cell control in the presence of IL-1β on day 10, *p* = 0.045) (Figure 1C,D).

Administration of TNF-α did not affect the levels of SOX9 expression, regardless of the time points evaluated (*p* = 0.090 compared with the cell control in the absence of cytokines) (Figure 1A,B versus Figure 1E,F). Overexpression of sox9 via rAAV led to increased levels of SOX9 expression (up to a 1.4-fold increase with respect to the cell control in the presence of TNF-α on day 10, *p* = 0.048) (Figure 1E,F). Notably, delivery of the vector via micellar carriers resulted in the highest levels of SOX9 expression (up to a 1.7-fold difference with respect to the cell control in the presence of TNF-α on day 1, *p* = 0.021) (Figure 1E,F).

Concomitant IL-1β/TNF-α application led to a decrease in the levels of SOX9 expression (up to a 1.2-fold difference with respect to the negative control in the absence of cytokines on day 10, *p* = 0.150) (Figure 1A,B versus Figure 1G,H). Significantly increased SOX9 levels were noted either using free rAAV-FLAG-hsox9 form (up to a 1.5-fold difference relative to the cell control in the presence of IL-1β/TNF-α on day 10, *p* = 0.019) (Figure 1G,H), or via delivery in PF68 or T908-based micelles (up to a 1.6-fold increase with respect to the cell control in the presence of IL-1β/TNF-α on day 1, *p* = 0.020) (Figure 1G,H).

### 3.2. Effects of rAAV-FLAG-hsox9/Polymeric Micelle Delivery on the Anabolic Activities of Human OA Chondrocytes in Inflammatory Conditions

We next investigated the effects of SOX9 overexpression on the deposition of type-II collagen and proteoglycans following rAAV-FLAG-hsox9 gene transfer via micellar vehicles in human OA chondrocytes monolayer cultures maintained in conditions of inflammation.

**Figure 2:**
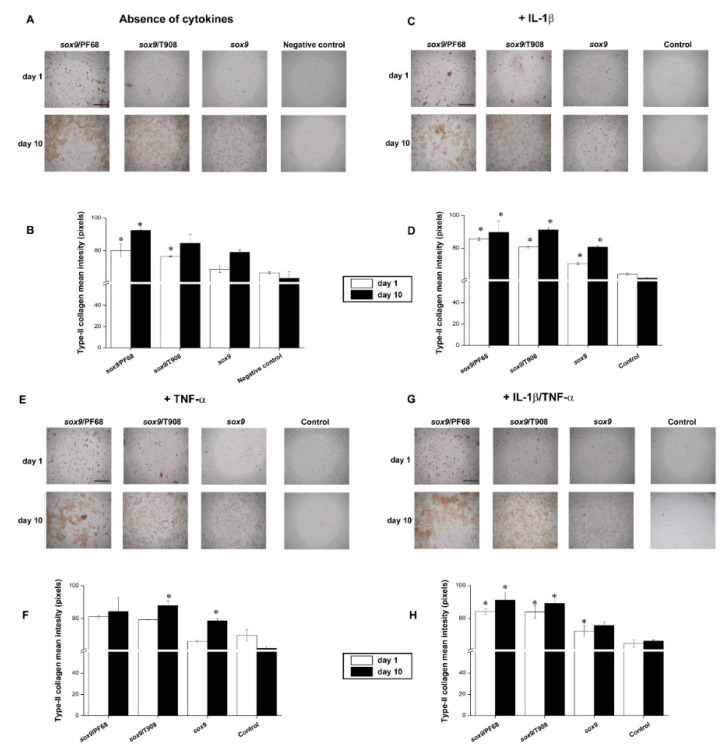
Remodeling activities in rAAV-FLAG-h*sox9*-transduced human OA chondrocytes using polymeric micelles. Cells in monolayer culture were directly transduced with the rAAV/polymeric micelles (**A**,**B**) or after pre-incubation for 4 h with IL-1β (10 ng/mL) (**C**,**D**), TNF-α (100 ng/mL) (**E**,**F**), or IL-1β/TNF-α (10/100 ng/mL) (**G**,**H**), as described in Figure 1 and in the Materials and Methods. The cultures were processed after 1 and 10 days to detect type-II collagen deposition by immunocytochemistry (magnification x10, scale bar 200 µm; all representative data) (**A**,**C**,**E**,**G**) with corresponding histomorphometric analyses (**B**,**D**,**F**,**H**), as described in the Materials and Methods. Control conditions included the absence of copolymer or vector treatment (negative control) and the application of free rAAV vector (positive control). * Statistically significant compared with the negative control at similar time points.

Administering of rAAV-FLAG-hsox9 significantly incremented type-II collagen deposition in the cells (up to a 1.3-fold increase with respect to the cell control in the absence of cytokines, *p* = 0.017) (Figure 2A,B). These levels increased over time, chiefly by delivery of the vectors via PF68 micelles (up to a 1.5-fold difference with respect to the negative control in the absence of cytokines on day 10, *p* = 0.040) (Figure 2A,B). Of note, these levels were higher than those achieved with free vector administration (up to a 1.2-fold difference compared with free rAAV-FLAG-hsox9 application on day 10, *p* = 0.011) (Figure 2A,B). Treatment with IL-1β decreased type-II collagen deposition (up to a 1.1-fold difference relative to the negative control in the absence of cytokines on day 1, *p* = 0.300) (Figure 2A,B versus Figure 2C,D). rAAV-FLAG-hsox9 application to IL-1β-treated chondrocytes significantly increased type-II collagen deposition, especially when using micelle-guided vector delivery (up to a 1.5-fold difference with respect to the cell control in the presence of IL-1β on day 10, *p* = 0.011; up to a 1.2-fold difference compared with free vector applying in the presence of IL-1β on day 10, *p* = 0.006) (Figure 2C,D). A similar tendency was noted when applying TNF-α alone or combined as a IL-1β/TNF-α co-treatment, showing modest decreases in type-II collagen deposition compared with cells kept in culture in the absence of cytokines (*p* = 0.290) (Figure 2A,B versus Figure 2E–H). Similarly, overexpression of SOX9 significantly increased type-II collagen deposition over time, particularly when providing rAAV-FLAG-hsox9 in micellar carriers (up to a 1.4-fold difference with respect to the cell control in the presence of TNF-α alone or as an IL-1β/TNF-α combination on day 10, *p* = 0.040) (Figure 2E–H).

**Figure 3:**
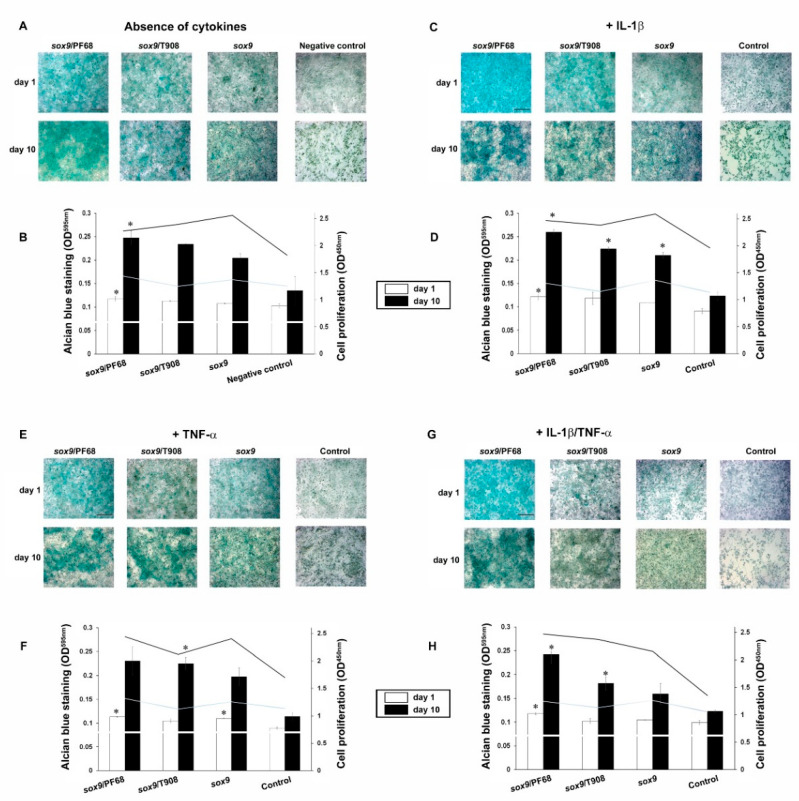
Biosynthetic activities in rAAV-FLAG-h*sox9*-transduced human OA chondrocytes using polymeric micelles. Cells in monolayer culture were directly transduced with the rAAV/polymeric micelles (**A**,**B**) or after pre-incubation for 4 h with IL-1β (10 ng/mL) (**C**,**D**), TNF-α (100 ng/mL) (**E**,**F**), or IL-1β/TNF-α (10/100 ng/mL) (**G**,**H**), as described in Figure 1 and Figure 2 and in the Materials and Methods. The cultures were processed at the denoted time points for Alcian blue staining (magnification x10, scale bar 200 µm; all representative data) (**A**,**C**,**E**,**G**) with spectrophotometric evaluations for cell proliferation and proteoglycan deposition following solubilization in 6 M guanidine hydrochloride (**B**,**D**,**F**,**H**), as described in the Materials and Methods. Control conditions included the absence of copolymer or vector treatment (negative control) and the application of free rAAV vector (positive control). * Statistically significant compared with the negative control at similar time points.

Overexpression of SOX9 in rAAV-FLAG-hsox9-transduced chondrocytes significantly increased the accretion of ECM-proteoglycans compared with untransduced cells (up to an 1.8-fold difference with respect to the negative control in the absence of cytokines on day 10, *p* = 0.030) (Figure 3A,B). Of note, delivery of rAAV-FLAG-hsox9 via micellar systems led to the highest proteoglycan deposition (up to a 1.2-fold increase with respect to free vector administering on day 10, *p* = 0.030) and proliferative index (up to a 1.4-fold increase with respect to the negative control in the absence of cytokines) (Figure 3A,B). Strikingly, treatment with IL-1β significantly decreased the deposition of proteoglycans and the cell proliferation ratio (up to a 1.2-fold difference with respect to the negative control in the absence of cytokines on day 1, *p* = 0.030) (Figure 3A,B versus Figure 3C,D). Additionally, rAAV-FLAG-hsox9-mediated transduction of IL-1β-treated chondrocytes prompted the restoration of proteoglycans, an effect more marked over time (up to a 1.7-fold increase when compared to the control in the presence of IL-1β on day 10, *p* = 0.038), exhibiting higher cell proliferation. Interestingly, providing rAAV-FLAG-hsox9 in micellar carriers led to the highest proteoglycan deposition (up to a 2.1-fold increase relative to the cell control in the presence of IL-1β on day 10, *p* = 0.006), reaching values that were higher than those reached with the free vector administration (up to a 1.3-fold difference with respect to free rAAV-FLAG-hsox9 application in the presence of IL-1β on day 10, *p* = 0.046) (Figure 3C,D). Treatment with TNF-α also decreased the deposition of proteoglycans (up to a 1.2-fold difference with respect to the negative control in the absence of cytokines on day 10, *p* = 0.203) and the cell proliferation index (Figure 3A,B versus Figure 3E,F). Transduction of TNF-α-treated chondrocytes with rAAV-FLAG-hsox9 significantly increased the cell proliferation and proteoglycan deposition, especially when the vectors were delivered via micellar systems (up to a 2-fold difference compared with the cell control in the presence of TNF-α on day 10, *p* = 0.010) (Figure 3E,F). Simultaneous IL-1β/TNF-α administration significantly decreased the deposition of proteoglycans (up to a 1.1-fold difference with respect to the negative control in the absence of cytokines on day 1, *p* = 0.011) and the cell proliferation rates (Figure 3A,B versus Figure 3G,H). Again, SOX9 overexpression increased the deposition of proteoglycans following IL-1β/TNF-α treatment, especially when the vectors were transferred via micellar vehicles (up to 2-fold difference relative to the cell control in the presence of IL-1β/TNF-α on day 10, *p* = 0.043; up to a 1.5-fold difference compared with free vector administration in the presence of IL-1β/TNF-α on day 10, *p* = 0.009) (Figure 3G,H). Likewise, genetic modification of chondrocytes via rAAV-FLAG-hsox9 resulted in an increased proliferation index (up to a 1.8-fold relative to the cell control in the presence of IL-1β/TNF-α on day 10, *p* = 0.001).

### 3.3. Effects of rAAV-FLAG-hsox9/Polymeric Micelle Delivery on the Viability Processes in Human OA Chondrocytes in Inflammatory Conditions

We finally examined the effects of SOX9 overexpression on the cell viability processes following rAAV-FLAG-hsox9 gene transfer via micellar systems in human OA chondrocytes monolayer cultures maintained in conditions of inflammation.

**Figure 4:**
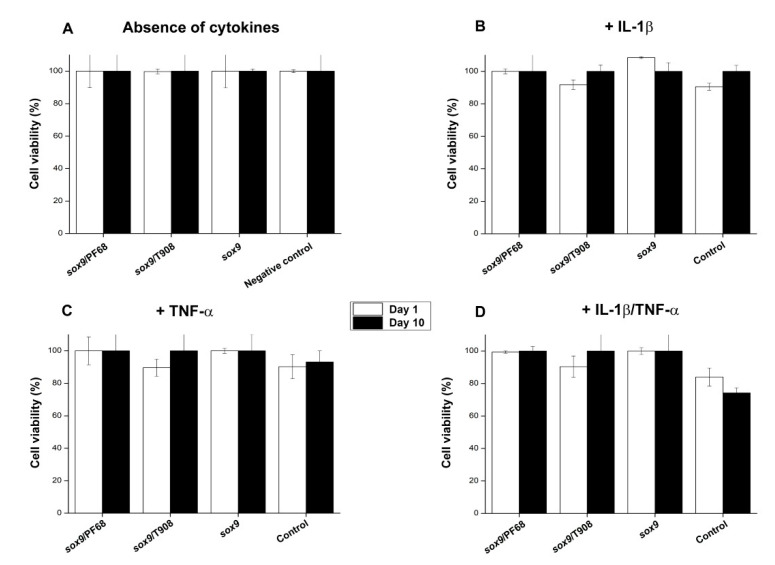
Cell viability in rAAV-FLAG-hsox9 modified human OA chondrocytes using micellar systems. Cell monolayer cultures were directly transduced with the rAAV/polymeric micelles (**A**) or after pre-incubation for 4 h with IL-1β (10 ng/mL) (**B**), TNF-α (100 ng/mL) (**C**), or IL-1β/TNF-α (10/100 ng/mL) (**D**), as described in Figure 1, Figure 2 and Figure 3 and in the Materials and Methods.

In concordance with our previous observations [23], no cytotoxic effects from none of the gene transfer procedures (polymeric vehicles, free vector supply) were noticed with respect to the control condition (*p* = 0.130) (Figure 4A). A similar tendency was evidenced when providing copolymer solutions in the absence of vector treatment (not shown). Moreover, while separate cytokine treatment resulted only in slight decreases in cell viability (~90%) (Figure 4B,C), concomitant administration of both cytokines led to higher toxicity especially in untransduced cells (~75% cell viability on day 10 in the negative control in the presence of IL-1β/TNF-α) (Figure 4D). Strikingly, overexpression of SOX9 led to higher cell viability indices in the presence of both cytokines (~100% compared with the cell control in the presence of IL-1β/TNF-α on day 10, *p* = 0.045) (Figure 4D).

## 4. Discussion

A potential means to counterbalance the disrupted cartilage homeostasis altered during OA disease is based on the correction of specific chondrocyte gene expression patterns [19]. Herein, transcription factors are critical mediators of cartilage metabolism prompting chondrogenesis in both physiologic and pathologic conditions [19]. Among them, SOX9 plays vital roles in the settlement of skeletal and cartilage formation [36] and the differentiation of chondrocytes [37]. Several studies have reported a decline in SOX9 expression in OA pathology [38,39]. Therefore, genetic adjustment of the levels of SOX9 expression may constitute a valuable strategy for re-equilibrating the disturbed balance characteristic of OA cartilage towards the synthesis of ECM compounds, affording the rescue of a native articular cartilage surface [19]. rAAV vectors are convenient carriers for efficiently and steadily targeting human OA chondrocytes [11,19] and avoiding the shortcomings and/or risks inherent to other types of vectors (short-term nonviral vectors, immunogenic adenoviral vectors, potentially tumorigenic retro-/lentiviral vectors) [40,41]. However, clinical administration of rAAV for OA treatments in patients may be hindered by the prevalence of circulating anti-AAV capsid antibodies in the subjects [42], especially in the synovial fluid from patients affected with joint disorders [20]. To overcome this hurdle, we evidenced the capability of PEO-PPO-PEO-based polymeric micelles (PF68 and T908) to efficiently and durably deliver rAAV vectors with increased stability and bioactivity to chondrocytes and mesenchymal stem cells (MSCs), affording protection against neutralizing antibodies [21,31]. Equally important, rAAV-mediated gene transfer of *sox9* via polymeric micelle delivery resulted in the remodeling of OA cartilage, with increased proteoglycan accumulation and cell proliferation in OA chondrocytes relative to free vector administration [23].

In light of these observations, the goal of the present study was to test the potentiality of these micellar nanocarriers to deliver the rAAV-FLAG-h*sox9* vector to human OA chondrocytes in an environment similar to that in OA, i.e., in the presence of pro-inflammatory IL-1β and TNF-α cytokines [4,5,24]. First, and in good concordance with our previous findings [23], the data indicate that the transfer of rAAV-FLAG-h*sox9* to human OA chondrocytes via polymeric micelles led to enhanced levels of SOX9 expression over time relative to free vector treatment. Of note, rAAV-FLAG-h*sox9* transduction of chondrocytes prompted elevated and sustained levels of SOX9 expression in cells treated with IL-1β, especially when the vectors were carried by the polymeric micelles. A similar trend was observed in the presence of TNF-α alone or combined with IL-1β (IL-β/TNF-α condition), showing that delivery of the vectors via micellar systems to the highest levels of SOX9 expression. Likewise, rAAV-FLAG-h*sox9*-mediated treatment in the presence of IL-β/TNF-α increased the levels of SOX9 expression. These results are in agreement with previous work reporting an increased rAAV-mediated modification of fibroblast-like synoviocytes in conditions of inflammation [43].

The results next indicate that rAAV *sox9* treatment led to significantly higher levels of type-II collagen deposition compared with untransduced controls, most particularly when the vectors were delivered via polymeric micelles, concordant with our previous work when providing rAAV-FLAG-h*sox9* to experimental human osteochondral defects [23] and with the pro-anabolic properties of this transcription factor [44,45]. Of further note, while administration of IL-1β to chondrocyte cultures decreased the levels of type-II collagen deposition, in agreement with previous findings [7], transduction with rAAV-FLAG-h*sox9* reversed such undesirable effects by increasing type-II collagen deposition in IL-1β-treated chondrocytes especially when providing the construct via polymeric micelles, expanding earlier work using lentiviral delivery of *sox9* [8]. In this regard, the use of rAAV provides strong advantages for clinical translation, as they do not carry the risk of insertional mutagenesis inherent to lentiviruses [9]. Similar observations were made following TNF-α treatment (alone or combined with IL-1β) and genetic modification via rAAV-FLAG-h*sox9*, with increased type-II collagen deposition especially using polymeric micelle-guided rAAV gene transfer. Also remarkably, SOX9 overexpression via rAAV was capable of reverting the inhibitory effects of the cytokines upon the deposition of proteoglycans and the proliferation index [7], especially when delivering the therapeutic construct in polymeric micelles, again expanding work with lentiviral gene delivery of *sox9* [8], and concordant with the pro-anabolic activities of the transcription factor [45]. Moreover, no detrimental effects were noted, regardless of the gene transfer method adopted, as previously described with rAAV [23], and SOX9 overexpression was again capable to counteract the cytotoxic effects of the cytokines by preserving the viability of the OA chondrocytes, in agreement with work highlighting the role of SOX9 to preserve chondrocyte survival [8].

## 5. Conclusions

The present study shows the potentiality of polymeric micelles as powerful rAAV controlled delivery systems to counteract the specific contribution of major OA-associated inflammatory cytokines in chondrocyte cultures. Here, we provide concrete evidence that encapsulation of an rAAV vector carrying a *sox9* sequence in such systems promotes significant SOX9 expression levels capable of increasing the deposition of major ECM components (type-II collagen, proteoglycans) and the cell survival processes in human OA chondrocytes while reversing their downregulation afforded by OA cytokines. While this work evidence the utility of such micellar systems to tackle the OA phenotype in chondrocytes in a 2D environment, work is currently ongoing to broaden this investigation at longer time points and to support the present findings when cells are embedded in their own pericellular matrix using an experimental model of osteochondral defect in situ [22,23] where the chondrocytes may also be influenced by interplay with subchondral bone cells that have key roles in OA development and progression. Overall, such observations show the effectiveness of polymeric micelles as rAAV controlled delivery systems in an inflammatory environment, making them attractive tools for the treatment for chronic inflammatory diseases like OA.

## References

[1] Hermann W., Lambova S., Muller-Ladner U. (2018). Current treatment options for osteoarthritis. Curr. Rheumatol. Rev..

[2] Loeser R.F., Goldring S.R., Scanzello C.R., Goldring M.B. (2012). Osteoarthritis: A disease of the joint as an organ. Arthritis Rheum..

[3] Poole A.R. (2012). Osteoarthritis as a whole joint disease. HSS J..

[4] Kapoor M., Martel-Pelletier J., Lajeunesse D., Pelletier J.P., Fahmi H. (2011). Role of proinflammatory cytokines in the pathophysiology of osteoarthritis. Nat. Rev. Rheumatol..

[5] Goldring M.B., Otero M. (2011). Inflammation in osteoarthritis. Curr. Opin. Rheumatol..

[6] Fernandes J.C., Martel-Pelletier J., Pelletier J.P. (2002). The role of cytokines in osteoarthritis pathophysiology. Biorheology.

[7] Hwang S.G., Yu S.S., Poo H., Chun J.S. (2005). C-jun/activator protein-1 mediates interleukin-1beta-induced dedifferentiation but not cyclooxygenase-2 expression in articular chondrocytes. J. Biol. Chem..

[8] Lu H., Zeng C., Chen M., Lian L., Dai Y., Zhao H. (2015). Lentiviral vector-mediated over-expression of sox9 protected chondrocytes from il-1beta induced degeneration and apoptosis. Int. J. Clin. Exp. Pathol..

[9] Schlimgen R., Howard J., Wooley D., Thompson M., Baden L.R., Yang O.O., Christiani D.C., Mostoslavsky G., Diamond D.V., Duane E.G. (2016). Risks associated with lentiviral vector exposures and prevention strategies. J. Occup. Environ. Med..

[10] Smith R.H. (2008). Adeno-associated virus integration: Virus versus vector. Gene Ther..

[11] Madry H., Cucchiarini M., Terwilliger E.F., Trippel S.B. (2003). Recombinant adeno-associated virus vectors efficiently and persistently transduce chondrocytes in normal and osteoarthritic human articular cartilage. Hum. Gene Ther..

[12] Cucchiarini M., Madry H., Ma C., Thurn T., Zurakowski D., Menger M.D., Kohn D., Trippel S.B., Terwilliger E.F. (2005). Improved tissue repair in articular cartilage defects in vivo by rAAV-mediated overexpression of human fibroblast growth factor 2. Mol. Ther..

[13] Hiraide A., Yokoo N., Xin K.Q., Okuda K., Mizukami H., Ozawa K., Saito T. (2005). Repair of articular cartilage defect by intraarticular administration of basic fibroblast growth factor gene, using adeno-associated virus vector. Hum. Gene Ther..

[14] Cucchiarini M., Orth P., Madry H. (2013). Direct rAAV sox9 administration for durable articular cartilage repair with delayed terminal differentiation and hypertrophy in vivo. J. Mol. Med..

[15] Cucchiarini M., Madry H. (2014). Overexpression of human IGF-I via direct rAAV-mediated gene transfer improves the early repair of articular cartilage defects in vivo. Gene Ther..

[16] Ortved K.F., Begum L., Mohammed H.O., Nixon A.J. (2015). Implantation of rAAV5-IGF-I transduced autologous chondrocytes improves cartilage repair in full-thickness defects in the equine model. Mol. Ther..

[17] Ulrich-Vinther M., Stengaard C., Schwarz E.M., Goldring M.B., Soballe K. (2005). Adeno-associated vector mediated gene transfer of transforming growth factor-beta1 to normal and osteoarthritic human chondrocytes stimulates cartilage anabolism. Eur. Cell Mater..

[18] Venkatesan J.K., Rey-Rico A., Schmitt G., Wezel A., Madry H., Cucchiarini M. (2013). rAAV-mediated overexpression of TGF-beta stably restructures human osteoarthritic articular cartilage in situ. J. Transl. Med..

[19] Cucchiarini M., Thurn T., Weimer A., Kohn D., Terwilliger E.F., Madry H. (2007). Restoration of the extracellular matrix in human osteoarthritic articular cartilage by overexpression of the transcription factor sox9. Arthritis Rheum..

[20] Cottard V., Valvason C., Falgarone G., Lutomski D., Boissier M.C., Bessis N. (2004). Immune response against gene therapy vectors: Influence of synovial fluid on adeno-associated virus mediated gene transfer to chondrocytes. J. Clin. Immunol..

[21] Rey-Rico A., Frisch J., Venkatesan J.K., Schmitt G., Rial-Hermida I., Taboada P., Concheiro A., Madry H., Alvarez-Lorenzo C., Cucchiarini M. (2016). PEO-PPO-PEO carriers for rAAV-mediated transduction of human articular chondrocytes in vitro and in a human osteochondral defect model. ACS Appl. Mater. Interfaces.

[22] Rey-Rico A., Venkatesan J.K., Schmitt G., Concheiro A., Madry H., Alvarez-Lorenzo C., Cucchiarini M. (2017). rAAV-mediated overexpression of TGF-beta via vector delivery in polymeric micelles stimulates the biological and reparative activities of human articular chondrocytes in vitro and in a human osteochondral defect model. Int. J. Nanomed..

[23] Rey-Rico A., Venkatesan J.K., Schmitt G., Speicher-Mentges S., Madry H., Cucchiarini M. (2018). Effective remodelling of human osteoarthritic cartilage by sox9 gene transfer and overexpression upon delivery of rAAV vectors in polymeric micelles. Mol. Pharm..

[24] Berenbaum F. (2013). Osteoarthritis as an inflammatory disease (osteoarthritis is not osteoarthrosis!). Osteoarthr. Cartil..

[25] Samulski R.J., Chang L.S., Shenk T. (1989). Helper-free stocks of recombinant adeno-associated viruses: Normal integration does not require viral gene expression. J. Virol..

[26] Samulski R.J., Chang L.S., Shenk T. (1987). A recombinant plasmid from which an infectious adeno-associated virus genome can be excised in vitro and its use to study viral replication. J. Virol..

[27] Venkatesan J.K., Ekici M., Madry H., Schmitt G., Kohn D., Cucchiarini M. (2012). Sox9 gene transfer via safe, stable, replication-defective recombinant adeno-associated virus vectors as a novel, powerful tool to enhance the chondrogenic potential of human mesenchymal stem cells. Stem Cell Res. Ther..

[28] Tao K., Rey-Rico A., Frisch J., Venkatesan J.K., Schmitt G., Madry H., Lin J., Cucchiarini M. (2016). rAAV-mediated combined gene transfer and overexpression of tgf-beta and sox9 remodels human osteoarthritic articular cartilage. J. Orthop. Res..

[29] Venkatesan J.K., Frisch J., Rey-Rico A., Schmitt G., Madry H., Cucchiarini M. (2017). Impact of mechanical stimulation on the chondrogenic processes in human bone marrow aspirates modified to overexpress sox9 via rAAV vectors. J. Exp. Orthop..

[30] Frisch J., Venkatesan J.K., Rey-Rico A., Schmitt G., Madry H., Cucchiarini M. (2014). Determination of the chondrogenic differentiation processes in human bone marrow-derived mesenchymal stem cells genetically modified to overexpress transforming growth factor-beta via recombinant adeno-associated viral vectors. Hum. Gene Ther..

[31] Rey-Rico A., Venkatesan J.K., Frisch J., Rial-Hermida I., Schmitt G., Concheiro A., Madry H., Alvarez-Lorenzo C., Cucchiarini M. (2015). Peo-ppo-peo micelles as effective rAAV-mediated gene delivery systems to target human mesenchymal stem cells without altering their differentiation potency. Acta Biomater..

[32] Vincenti M.P., Brinckerhoff C.E. (2001). Early response genes induced in chondrocytes stimulated with the inflammatory cytokine interleukin-1beta. Arthritis Res..

[33] Schuerwegh A.J., Dombrecht E.J., Stevens W.J., Van Offel J.F., Bridts C.H., De Clerck L.S. (2003). Influence of pro-inflammatory (IL-1 alpha, IL-6, TNF-alpha, IFN-gamma) and anti-inflammatory (il-4) cytokines on chondrocyte function. Osteoarthr. Cartil..

[34] Stanton L.A., Sabari S., Sampaio A.V., Underhill T.M., Beier F. (2004). P38 map kinase signalling is required for hypertrophic chondrocyte differentiation. Biochem. J..

[35] Woods A., Wang G., Beier F. (2005). Rhoa/rock signaling regulates sox9 expression and actin organization during chondrogenesis. J. Biol. Chem..

[36] Bi W., Deng J.M., Zhang Z., Behringer R.R., de Crombrugghe B. (1999). Sox9 is required for cartilage formation. Nat. Genet..

[37] Ikeda T., Kamekura S., Mabuchi A., Kou I., Seki S., Takato T., Nakamura K., Kawaguchi H., Ikegawa S., Chung U.I. (2004). The combination of sox5, sox6, and sox9 (the sox trio) provides signals sufficient for induction of permanent cartilage. Arthritis Rheum..

[38] Salminen H., Vuorio E., Saamanen A.M. (2001). Expression of sox9 and type IIa procollagen during attempted repair of articular cartilage damage in a transgenic mouse model of osteoarthritis. Arthritis Rheum..

[39] Aigner T., Gebhard P.M., Schmid E., Bau B., Harley V., Poschl E. (2003). Sox9 expression does not correlate with type II collagen expression in adult articular chondrocytes. Matrix Biol..

[40] Madry H., Cucchiarini M. (2013). Advances and challenges in gene-based approaches for osteoarthritis. J. Gene Med..

[41] Evans C.H., Huard J. (2015). Gene therapy approaches to regenerating the musculoskeletal system. Nat. Rev. Rheumatol..

[42] Calcedo R., Wilson J.M. (2013). Humoral immune response to AAV. Front. Immunol..

[43] Traister R.S., Fabre S., Wang Z., Xiao X., Hirsch R. (2006). Inflammatory cytokine regulation of transgene expression in human fibroblast-like synoviocytes infected with adeno-associated virus. Arthritis Rheum..

[44] Xie W.F., Zhang X., Sakano S., Lefebvre V., Sandell L.J. (1999). Trans-activation of the mouse cartilage-derived retinoic acid-sensitive protein gene by sox9. J. Bone Miner. Res..

[45] Furumatsu T., Matsumoto-Ogawa E., Tanaka T., Lu Z., Ozaki T. (2014). Rock inhibition enhances aggrecan deposition and suppresses matrix metalloproteinase-3 production in human articular chondrocytes. Connect. Tissue Res..

